# Editorial: Integrated clinical management and neurorehabilitation for lumbosacral spinal diseases

**DOI:** 10.3389/fneur.2025.1590602

**Published:** 2025-06-24

**Authors:** Moksada Regmi, Chenlong Yang

**Affiliations:** ^1^State Key Laboratory of Vascular Homeostasis and Remodeling, Department of Neurosurgery, Peking University Third Hospital, Peking University, Beijing, China; ^2^Center for Precision Neurosurgery and Oncology of Peking University Health Science Center, Peking University, Beijing, China; ^3^Peking University Health Science Center, Beijing, China; ^4^Henan Academy of Innovations in Medical Science (AIMS), Zhengzhou, China; ^5^Center for Oculocranial Pressure Instability Disorders (COPID), Zhengzhou, China

**Keywords:** lumbosacral spinal disease, treatment, neurorehabilitation, sphincter function, integrated medicine

The management of lumbosacral spinal diseases—spanning degenerative, oncologic, congenital, and traumatic pathologies—remains a complex challenge that demands a balance between innovation and validation. Recent studies highlight promising tools and therapies, but they also expose critical gaps in evidence, reproducibility, and clinical translation (1–5). This editorial summarizes key findings from the current Research Topic while emphasizing methodological limitations, unresolved questions, and actionable recommendations to steer future research toward meaningful patient-centered outcomes.

The integration of advanced imaging and computational modeling has undeniably improved diagnostic precision. One example from our own research is the development of an arbitrary-dimensional nerve root reconstruction MRI (ANRR-MRI) technique that offers a novel method to identify leakage points in sacral meningeal cysts, thereby enabling more targeted surgical interventions. While the reported 100 postoperative cyst resolution in 40 patients is notable, the lack of long-term follow-up data may raise concerns about recurrence rates and the durability of the results (Yang et al.) (6). Additionally, the homogeneity of the cohort (all patients were treated at a single center) limited the generalizability of the findings. Validation in diverse populations, including those with multifocal cysts or concurrent spinal abnormalities, is essential before ANRR-MRI can be widely adopted as a standard diagnostic tool. Similarly, Chen et al.'s machine learning model for predicting 1-year postoperative recovery in patients with lumbar disk herniation demonstrated the potential of artificial intelligence in personalized care (Chen et al.) (7). However, reliance on retrospective data from a single institution introduced inherent biases, and the model's performance metrics—while superior to other algorithms—lacked transparency in feature importance. Clinicians cannot trust a “black box” without understanding which variables (e.g., preoperative pain scores, comorbidities, or socioeconomic factors) drive predictions (8, 9). Future studies must prioritize interpretability and external validation across healthcare systems to avoid perpetuating biased algorithms.

Therapeutic advances, while innovative, frequently overlook cost-effectiveness and scalability. The study by Tan et al. on uniportal full-endoscopic (UFE) surgery for lumbar facet joint cysts under local anesthesia reported impressive pain relief and functional improvement in eight patients (Tan et al.) (10). However, the exclusion of patients with comorbidities or multilevel pathology, which is common in real-world practice, called into question the study's generalizability. Additionally, the small sample size and the absence of a control group (e.g., comparing UFE to conventional open surgery) also precluded definitive conclusions about UFE's superiority. Similarly, deep brain stimulation (DBS) for spinal cord injury (SCI) recovery has been praised for its neuromodulatory potential; however, the reviewed preclinical and clinical evidence remained fragmented. The majority of studies focus on acute injury models, ignoring chronic SCI cases in which neuroplasticity is diminished. Moreover, the emphasis on motor recovery overlooks potential autonomic benefits (e.g., bladder control), which are equally critical to patient quality of life. Without standardized protocols for DBS target selection, stimulation parameters, and rehabilitation integration, the therapy risks becoming a costly, unproven intervention rather than a scalable solution.

Although rehabilitation strategies are foundational to care, they often lack mechanistic rigor. The meta-analysis by Liu et al. advocated for suspension exercise training (SET) in lumbar disk herniation, citing significant improvements in pain and functional scores (Liu et al.) (11). However, high heterogeneity in outcomes (*I*^2^ = 86–92%) undermined confidence in its efficacy. Subgroup analyses suggested that SET combined with traditional Chinese medicine (TCM) yields better results than SET alone; however, the inclusion of TCM—a variable with its own unverified mechanisms—complicated the attribution of benefits. Are the improvements due to SET, TCM, or placebo effects? The study design did not disentangle these factors. Similarly, the GIGER MD biofeedback device for neurogenic bladder in children was shown to increase voiding capacity and reduce incontinence, but the 36-patient cohort lacked a control group, and the 6-month follow-up period was insufficient to assess sustained benefits. Rehabilitation research must adopt more rigorous methodologies, including sham-controlled trials and mechanistic studies, to isolate therapeutic effects from confounding variables.

The oncologic studies in this collection revealed the ongoing challenges of managing aggressive pathologies. The sacral chordoma cohort analysis identified lung metastasis as a key prognostic factor, with larger tumors and postoperative recurrence correlating with poorer survival (Shi et al.) (12). While these findings aligned with existing literature, the retrospective design and reliance on radiographic diagnoses (without biopsy confirmation in all cases) introduced potential misclassification bias. Furthermore, the study did not address the role of emerging therapies, such as targeted molecular agents, in the treatment of metastatic chordoma (13). Similarly, a case report of adult lumbosacral neuroblastoma highlighted the rarity of this malignancy but offered limited insight into optimal management. The decision to prioritize chemotherapy over radical resection reflects institutional bias rather than evidence-based consensus, emphasizing the need for collaborative registries to pool data and establish standardized guidelines for rare spinal tumors.

Given these limitations, we developed several recommendations. First, methodological transparency must be prioritized. Studies leveraging machine learning should publish their code and datasets to enable replication. Second, clinical trials—whether surgical or rehabilitative—must incorporate control arms and longer follow-up periods to distinguish treatment effects from the natural progression of the condition. Third, cost-effectiveness analyses are non-negotiable. Innovations such as UFE surgery and DBS will fail to translate into widespread practice if their benefits do not outweigh the economic burden on healthcare systems. Fourth, collaborative efforts to harmonize measures such as the ODI or JOA across institutions would reduce heterogeneity and enable meta-analyses with greater statistical power. Fifth, research should explicitly evaluate access barriers to advanced diagnostics (e.g., ANRR-MRI) or therapies (e.g., gene-targeted SMA drugs) in low-resource regions, rather than assuming scalability.

Finally, the field must move beyond symptom-centric outcomes. While pain and functional scores are important, patient-reported outcomes—such as mental health, social participation, and caregiving burden—are rarely measured. For example, while SMA therapies such as nusinersen improve motor function, their impact on familial stress and financial toxicity remains unstudied. Similarly, DBS studies for SCI focus on gait recovery but neglect bladder and bowel function, which patients often rank as higher priorities. A paradigm shift toward holistic, patient-defined endpoints is overdue.

In conclusion, the studies in this Research Topic illustrate both the promise and pitfalls of contemporary lumbosacral spinal care. While technological and therapeutic advances abound, their clinical value remains uncertain without critical appraisal, methodological rigor, and a commitment to equity. Researchers must resist the allure of novelty and instead embrace stability and patient partnership to ensure that progress translates into meaningful, accessible outcomes. The path forward requires not just smarter tools, but also wiser stewardship of innovation.
